# Reinventing mental health care in youth through mobile approaches: Current status and future steps

**DOI:** 10.3389/fpsyg.2023.1126015

**Published:** 2023-03-09

**Authors:** Laura Marciano, Sundas Saboor

**Affiliations:** ^1^Harvard T.H. Chan School of Public Health, Boston, MA, United States; ^2^Lee Kum Sheung Center for Health and Happiness and Dana Farber Cancer Institute, Boston, MA, United States

**Keywords:** mental health, youth, mobile, trace data, EMAS, chatbots, positive psychology

## Abstract

In this perspective, we aim to bring together research on mobile assessments and interventions in the context of mental health care in youth. After the COVID-19 pandemic, one out of five young people is experiencing mental health problems worldwide. New ways to face this burden are now needed. Young people search for low-burden services in terms of costs and time, paired with high flexibility and easy accessibility. Mobile applications meet these principles by providing new ways to inform, monitor, educate, and enable self-help, thus reinventing mental health care in youth. In this perspective, we explore the existing literature reviews on mobile assessments and interventions in youth through data collected passively (e.g., digital phenotyping) and actively (e.g., using Ecological Momentary Assessments—EMAs). The richness of such approaches relies on assessing mental health dynamically by extending beyond the confines of traditional methods and diagnostic criteria, and the integration of sensor data from multiple channels, thus allowing the cross-validation of symptoms through multiple information. However, we also acknowledge the promises and pitfalls of such approaches, including the problem of interpreting small effects combined with different data sources and the real benefits in terms of outcome prediction when compared to gold-standard methods. We also explore a new promising and complementary approach, using chatbots and conversational agents, that encourages interaction while tracing health and providing interventions. Finally, we suggest that it is important to continue to move beyond the ill-being framework by giving more importance to intervention fostering well-being, e.g., using positive psychology.

## Introduction

The COVID-19 pandemic has created a global crisis for mental health (Santomauro et al., 2021), with a 25% increase in depression and anxiety symptoms during the first year of the pandemic worldwide (World Health Organization, 2022). In 2022, in the United States, over 2.5 million young people had severe depression, with multiracial youth at the highest risk. However, 60% did not receive any mental health treatment (The State of Mental Health in America, 2022). Youth disparities in receiving treatment are wide: only 8.30% of Asian youth has seen a health professional or received medication for depression, followed by Black or African American (9.40%) and Hispanic youth (9.50%; The State of Mental Health in America, 2022). Notably, among people with health insurance coverage, 54% still did not receive help, pointing out a mismatch between health insurance coverage and access to mental health care (The State of Mental Health in America, 2022). In Europe, particularly Germany, Italy, The Netherlands, Romania, Bulgaria, Turkey, and Lithuania, the estimated prevalence of mental health disorders among young people ranged from 10 to 22% (Ravens-Sieberer et al., 2008; Kovess et al., 2015; Patalay et al., 2016). In most European countries, mental health conditions are the leading cause of disability among young people (Castelpietra et al., 2022). Simultaneously, mental health services have been disrupted, thus widening the treatment gap for mental health conditions. According to the World Health Organization (WHO), countries, on average, allocate less than 2% of the health care budget to mental health (World Health Organization, 2022). Hence, productivity costs and indirect losses to society far exceed healthcare costs.

Ongoing efforts to provide adequate support for young people’s mental health should follow the paths of transformation outlined by the WHO in July 2022, including augmenting value and commitment (e.g., by giving mental and physical health equal importance), reshaping the environment (e.g., including homes, schools, and communities in general), and strengthening mental health care (e.g., by making mental health affordable and accessible for all and promote a person-centered approach; World Health Organization, 2022). The need to reinvent mental health care is also in line with the last report of the National Institute of Mental Health (NIMH; National Institute of Mental Health, 2022). In particular, to address urgent needs after COVID-19 pandemic, NIMH has developed strategies, including computerized adaptive screening tools for suicide prevention, the launch of specific programs to reduce long-term disability of psychosis, and the increasing research efforts in mental health disparities using cutting-edge approaches like digital health technologies (National Institute of Mental Health, 2022). Overall, today, interventions to promote, monitor, and intervene on mental well-being require services and support that extend *beyond* clinical treatment and more attention should be allocated to overcoming factors that are stopping people from seeking help. These factors include poor quality of services, low health literacy levels, fear of stigma and discrimination, together with structural factors such as inaccessibility or high cost of mental health care and waiting lists (World Health Organization, 2022). In particular, young people search for low-burden services in terms of costs, time, flexibility, and accessibility (Reardon et al., 2017; Kahl et al., 2020; McGorry et al., 2022).

## A mobile approach to mental health

In 2022, 98% of U.S. teens have access to smartphones (Teens, 2022), and the same percentage can be found in Europe (Elavsky et al., 2022). In 11 countries (Croatia, Czech Republic, Germany, Estonia, Italy, Lithuania, Norway, Poland, Portugal, Romania, and Serbia), over 80% of children aged 9–16 use a smartphone to access the Internet at least once a day (Smahel et al., 2020). Hence, it is predictable that smartphone-based applications are promising approaches to inform, monitor, educate, and enable self-help in mental health care. Mobile approaches help diminish the stigma and discrimination related to mental health issues and overcome inaccessible or unaffordable therapies, especially among minorities and immigrants (Ashfaq et al., 2020; Drydakis, 2021). Mobile approaches are a promising avenue to bridge the gap between seeking help and accessing mental health resources in youth (Melcher et al., 2020; Onnela, 2021) offering alternative ways to (1) promote mental well-being by informing and educating the public, (2) support care by allowing professionals to provide remote assistance, and (3) improve people’s active engagement.

**Figure 1:**
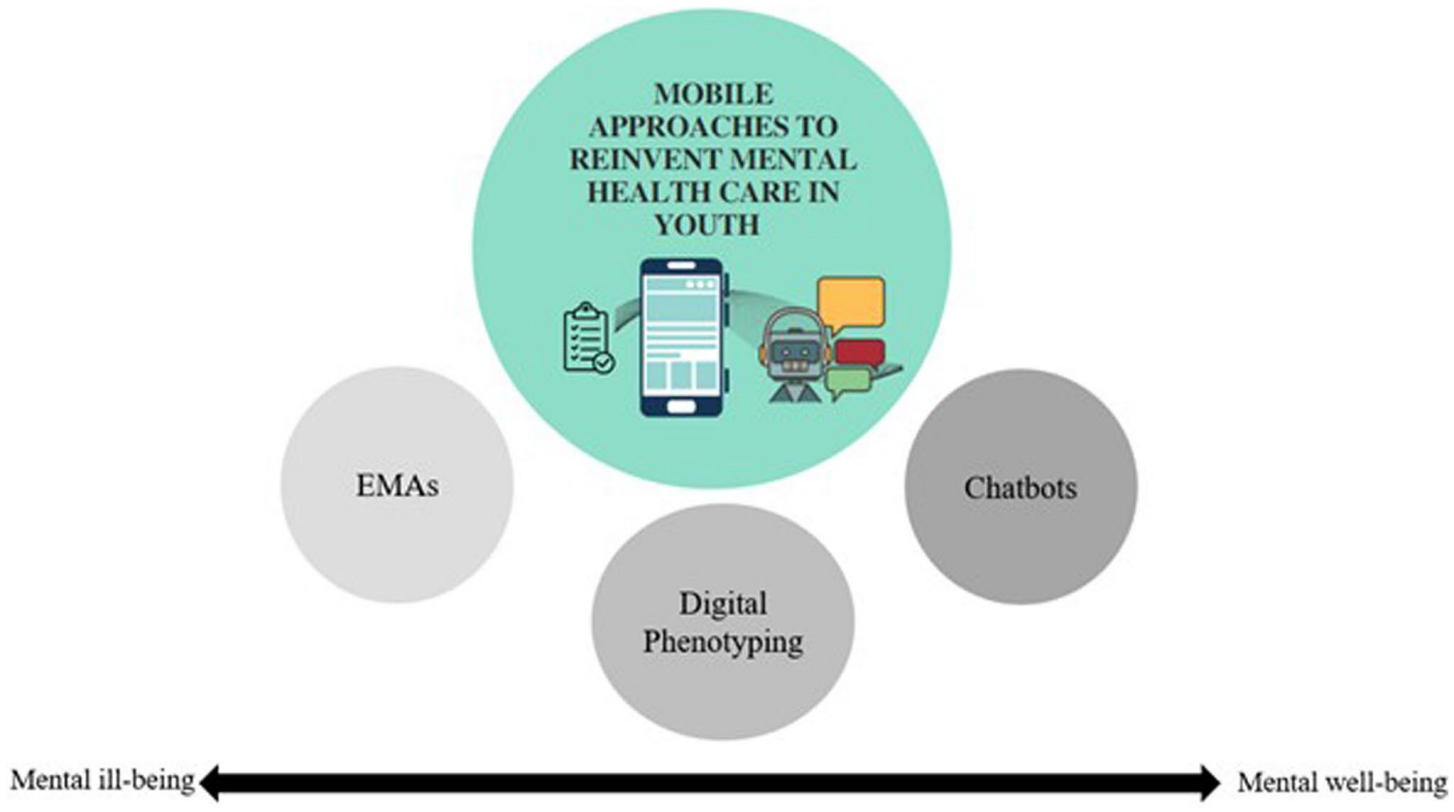
Overview of mobile approaches to reinvent mental health care in youth.

In the next paragraphs, we describe the research on mobile applications in the context of mental health care in youth by synthesizing results of existing reviews on data collected in passive and active ways using mobile approaches as well as through conversational agents such as chatbots (see Figure 1).

## Mobile approaches to passively and actively collect data

According to an ongoing umbrella review (Marciano, 2022), mobile assessments and interventions in youth include two types of collected data. First, passively collected trace data through mobile sensors, e.g., digital phenotyping, described as “the moment-by-moment quantification of the individual-level human phenotype *in situ* using data from personal digital devices, in particular, smartphones” (Torous et al., 2016, p. 3). It refers to the possibility of tracing and assessing mental health symptoms by automatically and uninterruptedly gathering data through smartphones and embedded sensors (e.g., GPS, digital camera, Wi-Fi, and Bluetooth), thus predicting human behaviors (e.g., physical activity, heart rate, temperature, and patterns of smartphone usage). Second, mobile approaches include actively monitoring subjective experiences using Ecological Momentary Assessments (EMAs), which involve the repetitive sampling of subjects’ current behaviors and moods at different moments of the day (Shiffman et al., 2008). EMAs encompass interpersonal interaction, self-monitoring, experience sampling, and ambulatory physiological assessment which relies on continuous or near-continuous recording rather than self-reporting (Shiffman et al., 2008). EMAs aim to minimize retrospective recall biases of traditional survey methods (Torous et al., 2014), maximize ecological validity (Russell and Gajos, 2020) by capturing human behavior in natural contexts, and study microprocesses (Shiffman et al., 2008) in real-time. Additionally, both trace data and EMAs, or their combination (Marciano et al., 2022), enable to model changes that happen at the individual level, thus allowing to overcome the Simpson’s paradox, for which the sign of a relationship observed in a population could become the opposite within the individuals (Kievit et al., 2013).

Overall, the richness of such approaches relies on the possibility of assessing mental health dynamically by extending beyond the confines of diagnostic criteria and integrating data from multiple channels. For example, smartphone apps are usually combined with wearable devices like actigraphy-based wrist-worn devices, biosensors, smartwatches, and digital rings, but also social media (Marengo and Montag, 2020), neuroimaging data (Camacho et al., 2021), and cortisol levels (Thunnissen et al., 2021).

Current research on trace and EMAs data in mental health mainly focused on depressive symptoms. For example, self-monitoring through EMAs may increase self-awareness of mood symptoms by tracing mood fluctuations (Beames et al., 2021). Additionally, much research has been conducted on bipolar disorders, schizophrenia, and psychosis (Baltasar-Tello et al., 2018; Sequeira et al., 2020) by collecting details on daily functioning, including social interactions and impairment experiences in relation to the clinical course of illness at an early or later stage (Benoit et al., 2020). Other current research focused on general psychopathology using the NIMH Research Domain Criteria (RDoC; Camacho et al., 2021; Thunnissen et al., 2021) to combine different levels of information, from genomics to behaviors, to describe mental health processes, problems, and illnesses. Also, information like sociability, including patterns of interaction and different behaviors like caffeine consumption, mind wandering, drinking alcohol, sleep, and physical activity, cognitive ability, are all information possible to relate to mental health (De Angel et al., 2022; Zarate et al., 2022). In rare cases, studies (de Vries et al., 2020) summarized the literature on mental well-being, including happiness, quality of life, life satisfaction, and positive affect.

Promises of using trace and EMA data in mental health care include the possibility of improving the self-efficacy and self-awareness of the patient and decision-making processes with the professionals. Additionally, it allows to cover the care gap also in low and middle-income countries that are increasingly digitalized, and to reach population minorities. Data integration from multiple sources also leads the ground for digital biomarkers, referring to the possibility of inferring biological information from digital trace data.

### Promises and pitfalls

We are enthusiastic about this view and we believe that collecting such richness of data would improve our understanding of human behaviors and mental health by considering variables and dynamics that were not possible to measure before. As in the field of genetics, “complex phenomena are likely to have many causes” (Götz et al., 2021, p.4). Although there has been a huge debate on how large an effect should be in order to have practical relevance for young people’s mental health (Funder and Ozer, 2019), the history of small effects already teaches (Elwood, 2006; Ittaman et al., 2014) us that also a tiny effect—when applied to a large-scale population and linked to a clinical outcome—is of practical relevance (e.g., the protective effect of aspirin on heart attacks). In the case of digital media use, also a small effect experienced by the population at large can lead to outcomes that are costly (e.g., higher depression rates which may turn in higher hospitalizations; Citrome et al., 2019); negative effects of social comparison on social media in youth may create more diagnoses of eating disorders (Sidani et al., 2016), higher distractibility, and cognitive load, which may turn in lower efficiency at work/school (Chen and Xiao, 2022; Stieger and Wunderl, 2022, etc.). That said, at large-scale, *also small effects matter*. How researchers report and interpret the small effect can make a difference. Coming back to the aspirin example, saying that taking the aspirin explains only a tiny effect (around 0.0011%) of the variance in the prevalence of heart attacks would not be very helpful. Instead, rephrasing this effect in terms of relative risk (i.e., who does not take aspirin has a doubled probability of having an heart attack with respect to those who took an aspirin) would give additional and practical information on the interpretation of the effects (Odgers et al., 2020). Indeed, complex psychological phenomena are likely to occur as a result of the interaction of tiny little effects, and small effects are most likely to be real in trying to explain human behaviors. Additionally, small effects can accumulate through time and at scale, which may be derived from different life domains accumulated during the years (Götz et al., 2021). To summarize, the use of EMAs and digital phenotyping would lead the ground for the exploration of small effects through the time and that would lead to test hypotheses of which we may not be even aware of. Hence, we encourage future researchers to describe longitudinal changes and processes, focusing on temporality (e.g., cause-effect mechanisms) and the interpretation of the small effects in describing human behavior.

Although, on one hand, digital phenotyping involves collecting massive amounts of individual data while creating a new category of health data and participation in risk assessment, on the other hand, existing ethical and regulatory frameworks for mental health care do not apply to digital phenotyping, creating the need to consider ethical, legal, and social implications (Potier, 2020). Indeed, digital phenotyping collects data about location, physical activity, mood, speech patterns, typing speed, call activity, social media usage, passive sensing data of GPS location, call logs for behaviorally phenotyping loneliness, and digital biomarkers (Baumgartner, 2021). However, the big data collected are still regarded as unclean and messy (Baumgartner, 2021). Today only a few research and healthcare organizations are collecting digital signals and these activities are largely exploratory (Huckvale et al., 2019). The data results are small-scale, partial, unstandardized, unlinkable, which results in multiple small data “silos” (Huckvale et al., 2019). These are insufficient for effective analysis due to noisy data. Also, to be useful, digital phenotyping must fit with established norms of quality and safety (Huckvale et al., 2019).

In some studies, digital phenotyping has been compared with gold-standard research methods to diagnose mental health disorders (Torous et al., 2019; Hays et al., 2020; Carpenter et al., 2021; Melcher et al., 2021; Moshe et al., 2021). However, more research should be carried out to highlight what EMAs and digital phenotyping can add to the information collected with gold-standard measures, in terms of explained variance in outcome variables and predicting power. Indeed, although data generated by digital phenotyping represent a striking parallel to gold-standard measures and mental status exams, there is still a need to compare the sensitivity, specificity, reliability, and variance of digital phenotyping with gold-standard measures (Onnela and Rauch, 2016). Hence, prospective longitudinal studies that include larger data sets from diverse populations are pivotal to instilling confidence in digital phenotyping among physicians, healthcare organizations, and hospitals to participate in the development of digital phenotyping to benefit patients and health consumers (Dlima et al., 2022). Some authors also claim that captured information through digital phenotyping only represents non-causal proxies or correlations as compared to actual causes of behaviors (Coghlan and D’Alfonso, 2021). As digital phenotyping studies advance, it increases the number of outcomes and predictors due to which many non-related outcomes and predictors will be found statistically significant by chance at 0.05 significance level (Barnett et al., 2018). If the family-wise error rate is controlled, it will lead to high false negatives and low specificity (Barnett et al., 2018). The error rates rely on tests being independent or having a specific dependence structure, while in digital phenotyping, many outcomes, and behaviors are highly correlated with unknown correlation structure, which decreases the sensitivity of a test (Barnett et al., 2018). Indeed, it is now crucial to move *from better data to better care*, by extrapolating meaningful information from complex data using, for example, machine learning techniques based on artificial intelligence. Also, the quality of the studies should be better evaluated by considering adherence rate and missing data handling. Last but not least, new data need new theories, however, theories are rarely mentioned in studies using digital phenotyping and EMAs.

## Use of chatbots

A new promising and complementary approach is using chatbots and conversational agents (Abd-Alrazaq et al., 2019; Vaidyam et al., 2019), which encourage interaction while tracing health and providing interventions. Indeed, people express their emotions and mood in different ways, and chatbots allow for detecting mental health problems and immediate interventions. Chatbots are computer programs capable of providing smart responses to user inputs by understanding natural languages using numerous Natural Language Processing (NLP) and generating appropriate natural human responses (Locke et al., 2021).

One review found that 39% of health chatbots focused on mental health issues (Milne-Ives et al., 2020). Chatbots may serve as the best means of increasing access to care (Vaidyam et al., 2021), showing empathy and ability to build relationships (Gaffney et al., 2019), and improving symptoms of depression and stress (Abd-Alrazaq et al., 2020). Currently, most mental health chatbots have been designed for depression and anxiety (Milne-Ives et al., 2020)among students (Milne-Ives et al., 2020; Klos et al., 2021), support children with autism (Cooper and Ireland, 2018), suicide risk, substance abuse (Prochaska et al., 2021), post-traumatic stress disorder (Han et al., 2021), and stress (Mauriello et al., 2021). They have also been used to enhance psychological well-being (Sweeney et al., 2021), self-compassion (Lee et al., 2019), and mindfulness (Denecke et al., 2021).

Previous reviews (Abd-Alrazaq et al., 2019; Bendig et al., 2019; Vaidyam et al., 2019; Boucher et al., 2021; Zhou et al., 2021) identified some essential attributes and features of chatbots that impact their performance and enhance psychological well-being, self-compassion, and mindfulness. As for their overall experiences and potential, for example, Vaidyam and colleagues described positive experiences with chatbots in regard to diagnostic quality, therapeutic efficacy, and acceptability (Vaidyam et al., 2021), finding that chatbots had a high diagnostic agreement with psychotherapists and moderate diagnostic agreement with psychology students and laypersons. Chatbots like Tess, Wysa, and SABORI, reduced self-identified depressive symptoms, elevated mood, and improved well-being, respectively. Assessment of acceptability showed positive adherence and satisfaction by participants. Abd-Alrazaq and colleagues summarized the perceptions and opinions of patients about chatbots for mental health, and key characteristics of the chatbots were usefulness, ease of use, responsiveness, understandability, attractiveness, trustworthiness, and enjoyability (Abd-Alrazaq et al., 2021). High usefulness was reported for practicing conversations in private places, psychoeducation, and feeling supported. The overall ease of chatbot use was rated as high. There were mixed or neutral perceptions and opinions about the responsiveness, i.e., verbal and non-verbal responses, however, chatbots were able to show friendly and emotional responses. Most of the studies concluded that participants believed that chatbots were trustworthy, enjoyable, and fun, satisfied with the chatbot content, and preferred talking to a chatbot as compared to a human for their healthcare needs. However, the chatbot attractiveness for participants was lower for reasons such as the lower quality of icons, buttons, font size, and appearance of the embodied chatbot. Additionally, in spite of all these benefits, chatbots have a lower diagnostic agreement with children and adolescents (Vaidyam et al., 2021), dialogues of rule-based chatbots are more difficult to continue since the patient feels not in control (Abd-Alrazaq et al., 2019), and chatbots cannot respond to difficult situations such as suicidal ideation beyond providing simple web search or helpline information (Vaidyam et al., 2019). Hence, overall, further research is needed to improve the psychotherapeutic content of chatbots and to investigate their usefulness through clinical trials (Bendig et al., 2019).

## Beyond ill-being

Last but not least, existing research on mobile applications for mental health care mainly focuses on ill-being outcomes, such as general mental health problems, depression, anxiety, internet use disorder, and psychosis. However, one review found that only two apps, i.e., HeadSpace and Calm, out of 27 existing apps for depression and anxiety, registered 96% of daily active users and 90% of monthly active users. Although they were not focused on positive psychology, they have a strong focus on prevention and well-being (Wasil et al., 2019) and have been searched only in the general population (O’Daffer et al., 2022). Considering that, in practice, daily active users heavily rely on apps that primarily use a preventative approach, we should now consider that complementary, preventative interventions are valuable. Also, there is great potential for a positive psychology equivalent in the research on mobile applications for mental health. As originally defined by the WHO, health is a “state of complete physical, mental and social well-being and not merely the absence of disease or infirmity” (World Health Organization, 1946, p. 1315). As established by positive psychology, “people also care about being happy, having a sense of meaning and purpose, being a good person, and having good relationships” (Wasil et al., 2019, p. 191). It is now crucial to consider that mental health exists on a complex continuum, ranging from ill-being to an optimal state of well-being. Mobile interventions based on positive psychology are still fairly nascent, however, conclusions from offline trials are promising (Bolier et al., 2013; Meyers et al., 2013; Kaplan et al., 2014). Only 1 month of intervention based on positive psychology showed effect sizes from small to moderate for mental well-being and depression (Bolier et al., 2013). Additionally, the effects persisted for up to 6 months. Thus, self-help interventions based on positive psychology “can serve as cost-effective mental health promotion tools to reach large target groups which may not otherwise be reached” (Bolier et al., 2013, p. 16) and possibly have a major impact on the population’s well-being. In particular, gratitude interventions showed improvements in mental health of the same size as those associated with clinical therapy techniques (Thunnissen et al., 2021), and they also improved physical health (Boggiss et al., 2020; Cousin et al., 2021). Additionally, the practice of loving-kindness meditation had long-lasting effects on the experience of positive emotions and personal resources (Cohn and Fredrickson, 2010). The use of these techniques is in line with a recent synergistic mindsets approach which proved to be effective in diminishing adolescents’ stress (Yeager et al., 2022).

## Discussion

To summarize, thanks to the flexibility and richness of trace and EMAs data, they are able to provide precious information on the individual, thus allowing to make personalized predictions and interventions and allowing the use of self-monitoring tools for personal and health management. This approach aligns with the recent field of “precision medicine” (Gameiro et al., 2018; Ginsburg and Phillips, 2018), which stresses the use of digital and health information to monitor well-being, prevent, and treat illness at a highly personalized level based on a prediction, prevention, personalization, and participation approach (Galas and Hood, 2009). Smartphone-based digital phenotyping gives new opportunities and challenges in collecting, analyzing, and interpreting data. By gathering different log and sensor information through mobile devices, it should be possible to study the individual and temporally dynamic behind well-and ill-being phenotypes, thus creating new markers to predict, monitor, and treat health-related problems. However, the interpretation of results coming from diverse data sources, likely explaining a small percentage of the outcome variance, and the comparison with gold standard methods remain an open issue, that should be addressed soon.

Future research needs to explore the combination of artificial intelligence chatbots, digital mental health interventions, and clinical support (Abd-Alrazaq et al., 2019, 2020; Bendig et al., 2019; Zhou et al., 2021). Research on the use of chatbots is still at the beginning, and new approaches, including NLP (Zhang et al., 2022) are now needed (Vaidyam et al., 2019) to understand the context and meaning of a statement, thus empowering proactive mental health care. A mental health chatbot must be empathic and able to establish a relationship with the user (Abd-Alrazaq et al., 2021). More randomized controlled trials and confirmatory studies of chatbots in the mental health context need to be performed. Also, considering that chatbots are prone to errors, these errors can be diminished by extensive training (Abd-Alrazaq et al., 2019; Boucher et al., 2021). The effectiveness of chatbots has mainly been explored in developed countries but might nevertheless also hold promise for developing countries (Abd-Alrazaq et al., 2019). Also, most of the current chatbot studies are primarily focused on (young) adults (Abd-Alrazaq et al., 2019). Only to a lesser extent, research focused on chatbots among youth (Koulouri et al., 2022). Future surveys and experiments should seek to examine youths’ well-being and mental health pre and post-chatbot use.

Overall, the use of trace and EMAs data together with chatbots can reinvent mental health care in youth, by bringing together powerful approaches able to integrate information from multiple channels and reach hardly reachable populations. The use of these mobile approaches would help diminish the mental health care gap and reduce the actual burden due to an increment in problems after COVID-19. We encourage future research to embrace these approaches, collecting data and running clinical trials to prove their efficacy and compare them to traditional methods.

## Data availability statement

The original contributions presented in the study are included in the article/supplementary material, further inquiries can be directed to the corresponding author.

## Author contributions

LM contributed to the conception of the paper and wrote the first draft of the manuscript. SS wrote some sections of the manuscript. All authors contributed to the article and approved the submitted version.

## Funding

The study was funded by the Swiss National Science Foundation (Grant No. P500PS_202974).

## Conflict of interest

The authors declare that the research was conducted in the absence of any commercial or financial relationships that could be construed as a potential conflict of interest.

## Publisher’s note

All claims expressed in this article are solely those of the authors and do not necessarily represent those of their affiliated organizations, or those of the publisher, the editors and the reviewers. Any product that may be evaluated in this article, or claim that may be made by its manufacturer, is not guaranteed or endorsed by the publisher.
